# Data on the optimization of the synthesis of green iron nanoparticles using plants indigenous to South Khorasan

**DOI:** 10.1016/j.dib.2018.11.030

**Published:** 2018-11-10

**Authors:** Abdollah Gholami, Rasoul Khosravi, Afshin Khosravi, Zahra Samadi

**Affiliations:** a Determination of health research center, Department of occupational Health Engineering, School of Health, Birjand University of Medical Sciences, Birjand, Iran; b Determination of health research center, Department of environmental Health Engineering, School of Health, Birjand University of Medical Sciences, Birjand, Iran

## Abstract

Green synthesis is a novel method for nanoparticle preparation, which is known as an environmentally friendly technique (Wang et al., 2017a, 2017b) [1], [2]. This research was carried out to investigate the use and efficacy of Barberry leaf, Elaeagnus angustifolia leaf, Saffron sepal, and Ziziphus jujube leaf extracts as agents for the synthesis of green iron nanoparticles (GINPs). The studied plants are among the native plants abundantly found in South Khorasan, Iran. The data also show the effect and role of important variables in green synthesis process including Fe to extract ratio, extract heating time, and length of time when Fe-extract solution was mixed under ultrasonic waves. The effects of the mentioned variables were measured by weighing the produced nanoparticle and determining the yield of the prepared nanoparticles. Based on the data, with decreasing Fe to extract ratio, the amount of produced GINPs was increased but the yield of the process decreased. Additionally, extract heating time and ultrasonic mixing time had a significant effect on GINPs yield. Based on the results of transmission electron microscopy (TEM) test, the size of GINPs in all of the plant extracts was about 40 nm and smaller.

**Specifications Table**

**Table:** 

Subject area	*Environmental engineering*
More specific subject area	*Chemistry*
Type of data	*Figures, images and text file*
How data was acquired	*Barberry leaf (BL), Elaeagnus angustifolia leaf (EAL), Saffron sepal (SS), and Ziziphus jujube leaf (ZJL) extracts were used to synthesis green iron nanoparticles (GINPs).*
	*The effect of Fe to solution ratio (1:1- 1:4), effect of the length of heating time during the extraction process (30 min -120 min), and the effect of the length of Fe-extract mixing using ultrasonic waves (30 min – 120 min) on the amount and yield of synthesized GINPs were investigated.*
	*Size and shape of GINPs were measured using Transmission Electron Microscopy (TEM) (Philips CM30 operating at 120 keV). In addition, ultrasonic waves (model Elmasonic E 30H) at a frequency of 37 kHz was used for mixing the solution and a pH meter (Sense Ion 378, Hack) was used for pH adjustment.*
	*The obtained data were analyzed using appropriate equation to calculate the yield of the synthesized GINPs*
Data format	*Raw, analyzed*
Experimental factors	*The data were obtained through assessing the effect of main optimization parameters on GINPs synthesis process.*
Experimental features	*The experiments were designed for the optimization of synthesis processes*
Data source location	*Birjand city, South Khorasan province, Iran*
Data accessibility	*All data are presented in this paper.*

**Value of the data**

• These data could be helpful for green synthesis of Iron nanoparticles using the plant extracts.
• The data on the synthesis processes optimization of the GINPs could give an insight of efficient optimizing parameters to other researchers interested in the green synthesis field.
• The data suggest that it is of great importance to optimize green synthesis process for every individual plant.
• The GINPs characterization date could provide an insight of the role of plant type on size and shape of the synthesized nanoparticles to other researchers.

## 1 Data

**Fig. 1:**
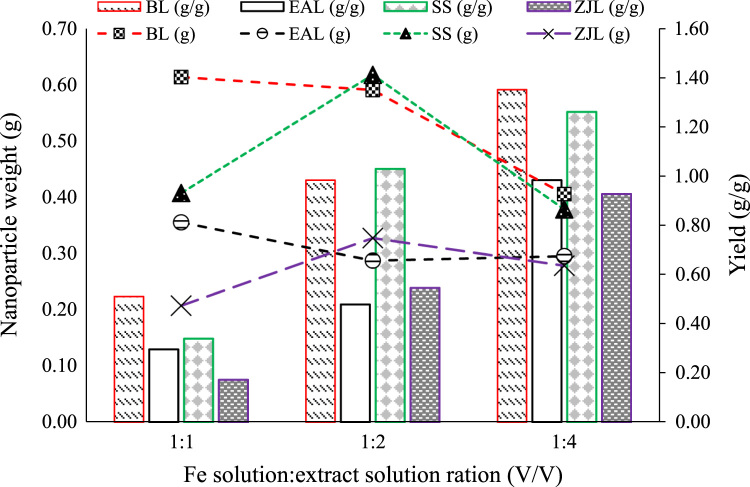
Effect of Fe solution to extraction solution ratio on amount and yield of obtained green nanoparticles.

**Fig. 2:**
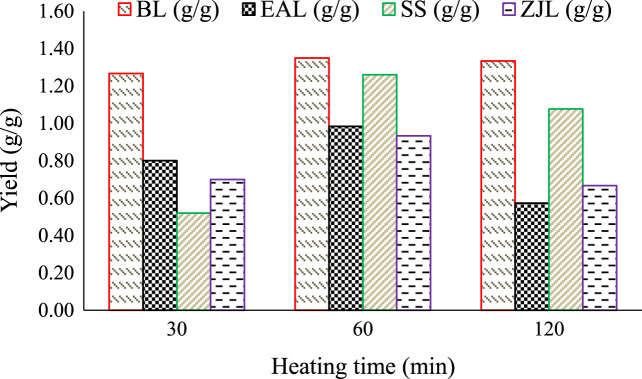
Effect of heating time at extraction process on yield of green nanoparticles.

**Fig. 3:**
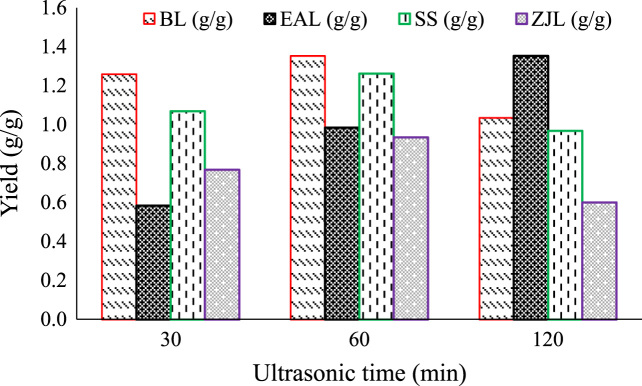
Effect of ultrasonic time at mixing process on yield of green nanoparticles.

**Fig. 4:**
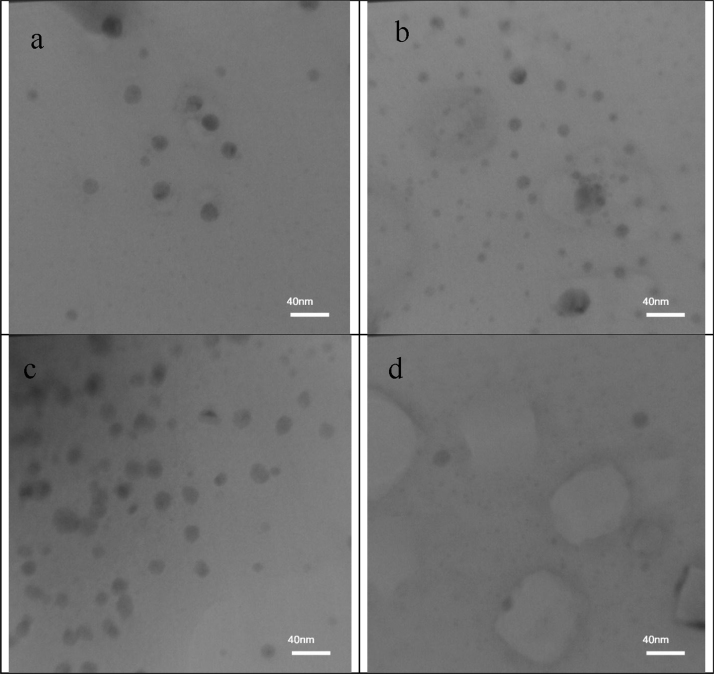
TEM images of the GINP synthesized from BL extraction (a), EAL extraction (b), SS extraction (c), and ZJL extraction (d).

The data presented in this paper illustrate the optimization of the process of green iron nanoparticles (GINPs) synthesis using the Barberry leaf, Elaeagnus angustifolia leaf, Saffron sepal, and Ziziphus jujube leaf, which are native plants abundantly found in South Khorasan province, East of Iran. The optimization parameters include Fe to extract ratio, extract heating time, and length of time when Fe-extract solution was mixed under ultrasonic waves. The data on optimization process are presented in Fig. 1, Fig. 2, Fig. 3. Transmission electron microscopy (TEM) was used to measure the size and shape of the synthesized GINPs. The obtained data are presented in Fig. 4.

## 2 Experimental design, materials, and methods

All the primary chemicals that were used for the synthesis process were purchased from Merck Co, Germany. The distilled water was used for the preparation of the solution. The pH of the solution was adjusted by adding NaOH and HCl 0.1 N.

For the synthesis of the GINPs, we used BL, EAL, SS, and ZJL extracts as the reducing and stabilizing agents [3], [4]. At first, the leaves and sepal of the mentioned plants were collected from agricultural area around of South Khorasan, Iran. Then, the collected raw agents were washed by distilled water several times to remove the dust and any impurities and dried at room temperature. Afterward, in order to investigate the effect of heating time, the extracts of BL, EAL, SS, and ZJL were prepared by dissolving 12 g of dry plants powder in 200 ml of distilled water and then heated for different times ranging from 30 to 120 min at 80 C on a heater magnetic stirrer [5], [6], [7]. After the precipitation for 1 h, the extracts were filtered by a vacuum pump. Then, a 0.1 M FeCl_2_ (H_2_O)_4_ was prepared by adding 3.98 g of solid FeCl_2_ (H_2_O)_4_ into 200 ml of deionized water. The effect of Fe to extract ratio (1:1, 1:2, 1:4) was investigated for all the plants. Accordingly, 100 ml of 0.1 M FeCl_2_ (H_2_O)_4_ was mixed into 100, 200, and 400 ml of the extract solution, and then the effect of mixing time was investigated to determine the optimum condition. Afterward, the mixed Fe and extract solution was transferred into an ultrasonic bath for forming a fine and efficient amount of GINPs. At this phase, we investigated the optimum ultrasonic time (30, 60, and 120 min) for mixing the solution. The formation of black colored precipitated material was the sign of GINPs synthesis. The synthesized GINPs were separated through the evaporation on a hot plate and collected through washing by distilled water for several times. Then, in order to dry the material, it was transferred into an oven and kept at 60 C for 12 h. The nanoparticles prepared at all the phases were weighed and the yield was calculated by Eq. (1):

(1) $$Y\;\left(\frac{g}{g}\right)=\frac{\mathrm{The\backslash amount\backslash of\backslash obtained}\;\mathrm{GINPs}\;(g)}{\mathrm{The\backslash amount\backslash of\backslash Fe}\;\mathrm{in\backslash the\backslash solution}\;(g)}$$

Size and shape of the synthesized green iron nanoparticles were analyzed by TEM.
