# Supplementary material for: Data on the optimization of the synthesis of green iron nanoparticles using plants indigenous to South Khorasan
Source: Data Brief. 2018 Nov 10;21:1779–83. doi: 10.1016/j.dib.2018.11.030 (PMC6251331; doi:10.1016/j.dib.2018.11.030)
Supplement: Supplementary file 1 — Transparency document [file mmc1.docx]

Dear editor(s)

Data in Brief

None of the authors of this paper have any conflicts of interest to declare.

Sincerely yours

Corresponding author

Rasoul Khosravi
